# Alzheimer’s and Related disorders Multicenter Archive (ARMA): a collaborative platform to advance ADRD diagnosis and treatment in Spain

**DOI:** 10.1002/alz.085813

**Published:** 2025-01-09

**Authors:** Ignacio Illán‐Gala

**Affiliations:** ^1^ Hospital de la Santa Creu i Sant Pau ‐ Biomedical Research Institute Sant Pau ‐ Autonomous University of Barcelona, Barcelona, Catalonia Spain

## Abstract

**Background:**

Alzheimer’s and related disorders (ADRD) represent a range of neurodegenerative conditions characterized by abnormal protein deposits in the brain. Despite advances, there is a need for enhanced diagnostic and treatment approaches that acknowledge the diversity of ADRD. This project introduces the Alzheimer’s and Related Disorders Multicenter Archive (ARMA), a collaborative platform with an advanced Electronic Data Capture (EDC) system linked to Electronic Medical Records (EMR) designed to refine ADRD diagnosis and natural history understanding, thus informing precision medicine.

**Specific Aims:**

ARMA aims to: 1) prove the feasibility of implementing an EDC‐augmented ARMA within two memory units; 2) Establish the clinical utility of biofluid and imaging biomarkers with real world data from diverse ADRD clinical profiles; 3) Develop the ARMA Trial Platform (ARMA‐TP) for clinical trials, hypothesizing enhanced, recruitment and retentionfor of diverse ADRD populations.

**Methodology:**

This four‐year project will recruit key personnel to integrate ARMA into clinical workflows, ensuring data integrity and GDPR compliance. The registry’s feasibility and the clinical utility of biomarkers will be evaluated, and disease progression will be tracked. Its advanced EDC system facilitates decentralized data collection, enhancing patient engagement and providing a scalable model for collaborative research. By its final year, ARMA‐TP will be launched to facilitate personalized treatment options in trials. Clinical‐biological profiles and recuittment data will be analyzed to describe biases in the recruitment process. Debiasing algorithms will be developed to ensure diverse recruitment and targeted recruitment campaigns will be designed to attract underrepresented ADRD profiles. We anticipate that the ARMA echosystem will reduce barriers to participation in both industry sponsored and industry‐independent trials, thereby improving the external validity of research findings.

**Impact:**

The ARMA project is set to transform collaboration within the Alzheimer´s Disease Barcelona Hub, impacting clinical practice and research in Spain. The project will be instrumental in improving the recruitment of a diverse and representative populations in clinical trials.

